# Exosomal and Non-Exosomal MicroRNAs: New Kids on the Block for Cancer Therapy

**DOI:** 10.3390/ijms23094493

**Published:** 2022-04-19

**Authors:** Shahzad Nawaz Syed, Bernhard Brüne

**Affiliations:** 1 Institute of Biochemistry I, Faculty of Medicine, Goethe-University Frankfurt, 60590 Frankfurt, Germany; b.bruene@biochem.uni-frankfurt.de; 2 Fraunhofer Institute for Translational Medicine and Pharmacology, 60596 Frankfurt, Germany; 3 German Cancer Consortium (DKTK), Partner Site Frankfurt, 60590 Frankfurt, Germany; 4 Cardio-Pulmonary Institute (CPI), 60590 Frankfurt, Germany; 5 Frankfurt Cancer Institute, Goethe-University Frankfurt, 60596 Frankfurt, Germany

**Keywords:** microRNA, cancer, macrophage, breast cancer, exosome, RNA therapeutics

## Abstract

MicroRNAs have been projected as promising tools for diagnostic and prognostic purposes in cancer. More recently, they have been highlighted as RNA therapeutic targets for cancer therapy. Though miRs perform a generic function of post-transcriptional gene regulation, their utility in RNA therapeutics mostly relies on their biochemical nature and their assembly with other macromolecules. Release of extracellular miRs is broadly categorized into two different compositions, namely exosomal (extracellular vesicles) and non-exosomal. This nature of miRs not only affects the uptake into target cells but also poses a challenge and opportunity for RNA therapeutics in cancer. By virtue of their ability to act as mediators of intercellular communication in the tumor microenvironment, extracellular miRs perform both, depending upon the target cell and target landscape, pro- and anti-tumor functions. Tumor-derived miRs mostly perform pro-tumor functions, whereas host cell- or stroma-derived miRs are involved in anti-tumor activities. This review deals with the recent understanding of exosomal and non-exosomal miRs in the tumor microenvironment, as a tool for pro- and anti-tumor activity and prospective exploit options for cancer therapy.

## 1. Introduction

Every crisis comes with an opportunity, which is also true for COVID-19. Treatment options for SARS-CoV-2 infections led to the early adoption of RNA therapeutics [1,2,3]. mRNA-based SARS-CoV-2 vaccine development and deployment gave the necessary impetus to drive RNA-based therapeutics for other life-threating diseases, including cancer [4] (ClinicalTrials Identifier: NCT02410733; NCT02410733; NCT02316457). The goal of the SARS-CoV-2 vaccine was to deliver an RNA-based viral message to mount antiviral host responses. In cancer, endogenous RNA (mRNA or small non-coding RNA) responses can be modulated with RNA therapeutics, as discussed in this review.

The genesis of unicellular and multicellular organisms shares a common trait that is crucial for their success of life, which is the propagation of signals to the environment, including cellular environments. The uncanny perfection with which cells communicate with their environment using chemicals, including nucleic acids, is responsible for the evolution of complex life forms, and dysregulation therein plays an important role in their diseases. An established tumor acts as a multicellular parasitic organism within its host and relies on this communication. The tumor microenvironment consists of a plethora of soluble factors, either derived from the tumor cell or stroma, which take part in intercellular communication. These factors play a pivotal role in tumor growth and dissemination. Amongst these factors, small nucleic acids such as microRNAs (miR) play a prominent role in the quest of regulatory factors for cancer therapy since virtually all biological pathways are under the control of miRs as most mRNAs contain at least one functional miR target site [5,6]. Furthermore, in the human genome, protein coding transcripts only account for about 2%, whereas non-coding RNAs (including miRs) correspond to about 98% of the total genome [7,8], strongly arguing for the importance of miRs in the regulation of biological processes. miRs are small non-coding RNAs of 20–25 nucleotides that post-transcriptionally regulate the expression of mRNAs [9]. Each mRNA may harbor multiple miR target sites, and multiple miRs can target a single mRNA [10]. In heathy cells and stroma, miRs systematically regulate the molecular RNA network, whereas in tumor cells, dysregulated miRs disrupt the precision of this network, leading to cancer progression [11]. Tumors as well as stroma release miRs in the close milieu for paracrine action. These miRs are packaged in different biochemical compositions to protect them from RNase degradation. Broadly, released miRs can be classified into two categories, exosomal and non-exosomal. Non-exosomal miRs, in principle, are associated with stabilizing factors such as argonaute protein (AGO) and other RNA binding proteins.

Classically, exosomal and non-exosomal miRs were differentiated using RNase to digest non-protected miRs. However, this differentiation has a serious limitation as it cannot discriminate AGO-protected miRs from exosomal miRs [12]. Furthermore, studies have shown that miRs are sorted and released mostly in the exosomal compartment, partly due to evolutionary pressure to preserve miRs from RNase action, or in part due to the secretory mechanism of donor cells [13]. Nevertheless, the tumor microenvironment exerts a ‘survival pressure’ on the host as well as tumor cells [14,15,16]. These adaptations may compel non-exosomal transfer of miRs in the tumor microenvironment. A desperate attempt of tumors to hijack host immune responses also comprises the secretion of non-exosomal miRs, taken up by phagocytes in the close vicinity [17,18]. However, reports on non-exosomal miRs are in the minority and are scarcely described in the literature. Nevertheless, non-exosomal miRs play an equally important role in cancer progression and hence in cancer therapy.

## 2. Exosomal MiRs in Cancer

Classification of extracellular vesicles (EVs), such as the exosome, is a complex process and is based on size and composition. Studies have identified at least two distinct subpopulations of exosomes, e.g., Exo-L (large, 90–120 nm) and Exo-S (small, 60–80 nm), which show distinctive biophysical and molecular properties as well as non-membranous “exomeres” (~35 nm) [19]. These exomeres are the predominant extracellular particles released by most studied cancer cells [19]. These exosome species show diverse organ distribution patterns and cargo content, which suggests specific biological functions [20]. For the sake of simplicity, we refer to these exosome species that encapsulate miR as “exosome” in this review.

**Figure 1:**
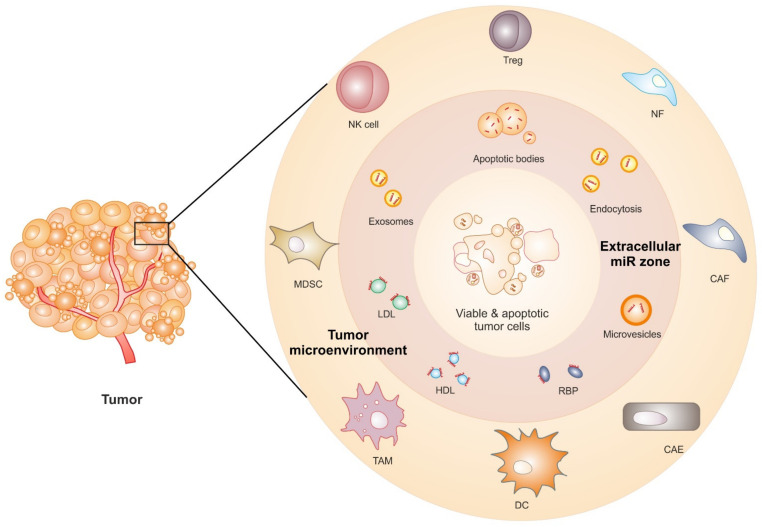
Extracellular miRs in the tumor microenvironment. The tumor microenvironment consists of viable and apoptotic tumor cells, along with stroma cells such as tumor-associated macrophage (TAMs), dendric cells (DC), cancer-associated endothelial cells (CAE), cancer-associated fibroblast (CAF), normal fibroblast (NF), regulatory T cells (Treg), natural killer (NK) cells, myeloid-derived suppressor cells (MDSC), etc. These cells crosstalk with extracellular miRs from the ‘extracellular miR zone’ in various forms and compositions, such as apoptotic bodies, endocytosis, and micro-vesicles, bound with RNA binding proteins (RBP), high-density lipoprotein (HDL), low-density lipoprotein (LDL), or exosomes.

Release and uptake of EVs are one of the most important modes of intercellular communication, including in the tumor microenvironment (Figure 1). Healthy or apoptotic cells release EVs, including exosomes, which are 30–150 nm in diameter and are derivatives of the multivesicular endosome pathway [21,22,23]. Exosomes contain bioactive molecules such as DNA, RNA, non-coding RNA, and miRs. Along with these nucleic acids, exosomes also contain proteins such as RNA binding protein—AGO, receptors, transcription factors, matrix protein, and lipids that alter the biochemical response of a recipient cell [24,25,26,27]. From an immunology standpoint, exosomes are characterized by the presence of tetraspanins (CD63, CD9, CD81), syndecan, heat shock proteins (HSPs), ALG-2-interacting protein X (Alix), and tumor susceptibility gene 101 (TSG101) [28,29,30]. The presence of these proteins not only aids in the identification of exosomes in biological samples but also highlights the complexities and presents exosomes as putative therapeutic targets or drug carriers for cancer, as discussed below. Furthermore, patient-derived circulating exosomes have been linked with relapse or metastasis and thus could serve as diagnostic markers as well as therapeutic targets [31].

Exosomal miR can be exchanged between the tumor and stroma and repress gene expression [32,33]. However, it is quite challenging to characterize the functionality of exosomal miR in recipient cells because the recipient cells usually express the same endogenous miR to varying degrees. This major hurdle precludes an accurate evaluation of the transferred miRs in the recipient cells and their functionality in the tumor microenvironment. A summary of the major composition and mode of extracellular miRs is depicted in Figure 1.

### 2.1. Tumor-Derived miRs

**Figure 2:**
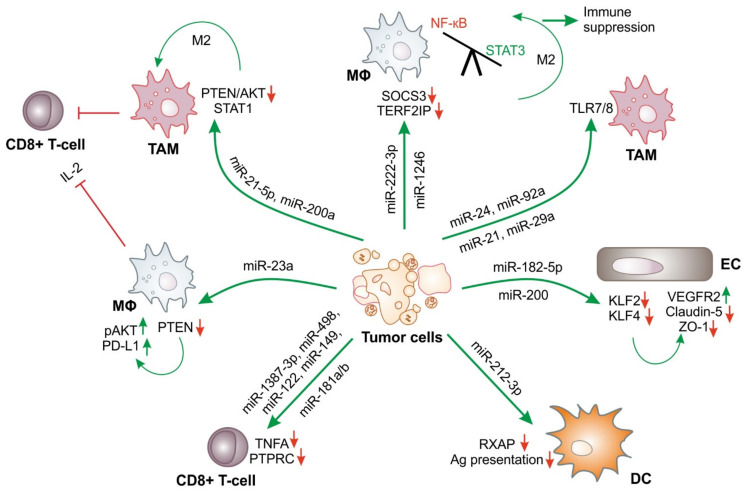
Tumor exosome-derived miRs in the tumor microenvironment. Exosomal miRs are involved in nearly all aspects of tumor immunity in the tumor microenvironment. See Section 2.1 for details. M2, alternative (M2) polarization; Ag, antigen; Mϕ, macrophage. ↓ = Downregulation and ↑ = upregulation. Green arrows are pro-tumoral and red arrows are anti-tumoral.

One of the most important functions of tumor-derived miRs is to dampen immune responses. There is ample evidence in the literature that tumor-derived exosome-contained miRs, once taken up by target immune cells, suppress anti-tumor immune responses. One of the ways how pancreatic cancer cells evade anti-tumor immunity is via secreting exosomal miRs. Exosome-containing miR-212-3p downregulates the expression of major histocompatibility complex (MHC)-II by blocking the MHC-II transcription factor regulatory factor X-associated protein (RFXAP) in dendritic cells (DCs) (Figure 2), thereby inducing immune tolerance [33]. On the other hand, colorectal cancer (CRC) cells secrete exosomes containing miR-21-5p and miR-200a. These exosomes are taken up by tumor-associated macrophages (TAMs), and exosomes containing miRs downregulate phosphatase and tensin homolog deleted on chromosome 10 (PTEN)/v-akt murine thymoma viral oncogene homolog (AKT), suppressor of cytokine signaling (SOCS)1/signal transducer and activator of transcription (STAT)1 (Figure 2). Thereby, TAMs are programmed towards the tumor-promoting M2 phenotype, showing enhanced expression of programmed death-ligand 1 (PD-L1). These PD-L1^+^CD206^+^ macrophages then decrease CD8^+^ T cell activity and increase tumor growth [34]. Similarly, epithelial ovarian cancer (EOC)-derived exosomal miR-222-3p downregulates SOCS3 expression in macrophages, which promotes STAT3-mediated M2 polarization, thereby generating an immunosuppressive tumor microenvironment (Figure 2) [35]. Exosomes from hypoxic glioblastoma (GBM) cells induce proangiogenic programming of endothelial cells and GBM cell proliferation [36]. Furthermore, it was shown that hypoxic GBM-derived exosomal miR-182-5p targets Krüppel-like factor (KLF) 2 and KLF4 in endothelial cells (Figure 2), which increases the promoter activity of vascular growth factor receptor (VEGFR)2 and decreases the expression of tight-junction proteins, such as claudin-5, occludin, and ZO-1. Ultimately, this promotes tumor angiogenesis and growth [37]. Similarly, exosomes from hypoxic glioma cells are phagocytized by macrophages and reprogram them to the alternatively activated M2 type. Glioma exosomes contain hypoxia-enriched miR-1246, which targets telomeric repeat-binding factor 2-interacting protein 1 (TERF2IP). Downregulation of TERF2IP skews the balance of macrophage polarization towards M2 by inhibiting nuclear factor (NF)-κB signaling and activating the STAT3 pathway (Figure 2) [38]. Immunosuppressive modulation of macrophages can also be achieved by hepatocellular carcinoma cell (HCC)-derived exosomes under stress conditions such as endoplasmic reticulum stress (triggered by tunicamycin), upon phagocytosis. HCC exosomes contain miR-23a, which downregulates PTEN expression and induces phosphorylation of AKT and PD-L1 in macrophages (Figure 2). These reprogrammed macrophages then decrease CD8^+^ T cell activation by interfering with interleukin (IL)-2, which increases T cell apoptosis [39]. This seem to be a common pattern of how tumor cells dampen anti-tumor immune responses by secreting regulatory miRs in exosomes. Vignard et al. also demonstrated that melanoma cell-derived exosomes are taken up by CD8^+^ T cells and dampen anti-tumor T cell responses by downregulating TNF-α, reducing granzyme B secretion and attenuating TCR signaling. Melanoma-derived exosomes contain high amounts of miR-1387-3p, miR-498, miR-122, miR-149, and miR-181a/b. These miRs target *TNFA* and *PTPRC* genes and dampen CD8^+^ T cell responses towards melanoma (Figure 2) [40].

Inflammatory conditions favor tumor growth and migration. Tumor-derived factors also alter the tumor microenvironment in a way that low-grade inflammation persists. Cancer cells also use miRs to achieve these objectives. Casadei et al. reported that liposarcoma cell-derived exosomes are rich in miR-24 and miR-92a. These miRs stimulate the production and release of proinflammatory IL-6 from TAMs via toll-like receptor (TLR)7/8 activation and NF-κB signaling (Figure 2). The release of IL-6 aids in the proliferation, migration, and invasion capacity of liposarcoma cells [41]. Similarly, in HCC, tumor-derived exosomes deliver miR-1247-3p to fibroblasts and reprogram them to cancer-associated fibroblasts (CAFs) in the lung pre-metastatic niche by directly targeting beta-1,4-galactosyltransferase 3, that provokes β1-integrin-NF-κB signaling [42]. In turn, activated CAFs then promote cancer progression by creating a local inflammatory milieu by the release of IL-6 and IL-8. Yet another way in which tumor-derived exosomal miR activates NF-κB signaling is by triggering TLRs to create a pro-tumoral inflammatory microenvironment that fosters tumor growth and metastasis. Exosomal miR-21 and miR-29a from lung cancer cells activate mouse TLR7- and human TLR8-mediated NF-κB activation in immune cells and the production of proinflammatory IL-6 and TNF-α to promote lung cancer and metastasis in mice [43].

Exosomes derived from cancer cells are not only enriched in miRs but also contain a complete miR cargo, including pre-miRs, proteins involved in miR biogenesis and function, such as the RISC (RNA-induced silencing complexes) loading complex (RLC), Dicer, transactivation response element RNA-binding protein (TRBP), and AGO2, and thereby can process pre-miR to generate mature miRs [44,45]. A study shows that exosomes of prostate cancer cells contain miR-125b, miR-130, miR-155, as well as H-*ras* and K-*ras* mRNAs, and Rab proteins (Rab1a, Rab1b, and Rab11a). When these exosomes are exposed to adipose stem cells, it enhances prostate tumor formation in vivo by inducing genetic instability, mesenchymal-to-epithelial transition, and oncogenic transformation [45]. The oncogenic transformation is associated with miR-mediated downregulation of tumor suppressor genes, e.g., large tumor suppressor kinase 2 and programmed cell death 4 (PDCD4).

Several tumor-derived miRs take part in tumor dissemination in a wide variety of cancers in various ways. Breast cancer exosome-derived miR-22 suppresses pyruvate kinase and subsequent glucose uptake in the lungs, which promotes metastasis [46]. Exosomal miR-23a is significantly enriched in transforming growth factor (TGF)-β1-treated human lung adenocarcinoma cells and is involved in epithelial-to-mesenchymal transition (EMT) [47], which is directly proportional to tumor invasion and metastasis [48]. miR-499a-5p is upregulated in highly metastatic lung cancer-derived exosomes and enhances cell proliferation, migration, and EMT by targeting the rapamycin (mTOR) pathway, which could be inhibited by antagomiR-499a-5p [49]. Inversely, tumor exosome anti-metastatic miR-192 significantly attenuates tumor metastasis by suppressing the expression of angiogenic factors such as IL-8, intercellular cell adhesion molecule (ICAM), and C-X-C motif chemokine ligand 1 (CXCL1) [50]. Interestingly, miR-192 shares the seed sequence with other family members such as miR-215, however both of these miRs induce an invasive activity only in vitro and differ in their functions in vivo [50]. This suggests that uncharacterized cell-specific factors are required for miRs to modulate cellular functions in vivo, which should be taken into consideration for the selection of miRs as putative cancer therapeutic targets. No doubt, despite the same seed sequence, miR family members have different targetomes, which might explain their cell-specific and context-dependent actions in vivo.

The plasticity of cancer cells may also be attributed, at least in part, to exosomal miRs. Strongly metastatic breast cancer cells secrete exosomal miR-200, which enhances EMT and metastasis of otherwise weakly metastatic breast cancer cells in close proximity [51]. Exosomes from highly metastatic HER2^+^ MCF10CA1a and basal-A TNBC cells deliver miR-200 to poorly metastatic basal-B TNBC MDA–MB-231 cells to promote lung metastasis in mice [51]. Breast cancer cell-derived exosomes suppress endothelial tight-junction zonula occludens 1 (ZO-1) expression via the action of miR-105, resulting in increased metastasis by enhancing vascular permeability and impairing the integrity of blood vessels in vivo (Figure 2) [52]. Similarly, miR-663b expression is elevated in cervical cancer tissue and is secreted in exosomes by cancer cells. miR-663b transferred to endothelial cells via exosomes downregulates its target vinculin, which plays a crucial role in focal adhesion formation, cell proliferation, and modulating the actin cytoskeleton. miR-663b-mediated downregulation of vinculin promotes the growth, metastasis, and angiogenesis of implanted tumors in mice [53]. Furthermore, radiation therapy enhances exosomal miR-7-5p levels by human bronchial epithelial cells, which induces bystander cell autophagy by targeting EGFR/Akt/mTOR signaling pathways [54]. Gastric cancer (GC) cell-derived exosomes contain miR-1290, which suppresses T cell activation by targeting the grainyhead-like 2 (GRHL2)/zinc finger E-box binding homeobox 1 (ZEB1) pathway, thereby provoking immune escape [55]. In GC cell-derived exosomes, miR-155 has been implicated in tumor growth and angiogenesis [56]. GC exosome-derived miR-155 directly targets c-MYB and increases the expression of VEGF, thereby promoting the growth, metastasis, and vascular cell tube formation [56]. Pancreatic tumor-derived exosomal miR-212-3p inhibits MHC-II by downregulating the expression of regulatory factor X-associated protein (RFXAP), which promotes the immune tolerance of DCs (Figure 2) [33].

In essence, tumor-derived exosomal miRs have been shown to touch upon almost all hallmarks of cancer [48]. It is interesting to note, especially in the context of developing RNA-based cancer therapeutics, that most of the extracellular miRs in the tumor microenvironment are identified in already established tumors or in tumors that already escaped anti-tumor immunity. Even as biomarkers, these miRs are released in the circulation when the tumor is already established. Early detection of oncogenic miRs may revolutionize RNA therapeutic approaches of cancer since established tumors are multifactorial and targeting a single miR may not be sufficient to cease the disease. Moreover, the extracellular miR landscape might vary in different stages of cancer and therapeutic targets or a biomarker identified from late-stage cancer might not serve as the best candidate.

### 2.2. Stroma-Dervied miRs

**Figure 3:**
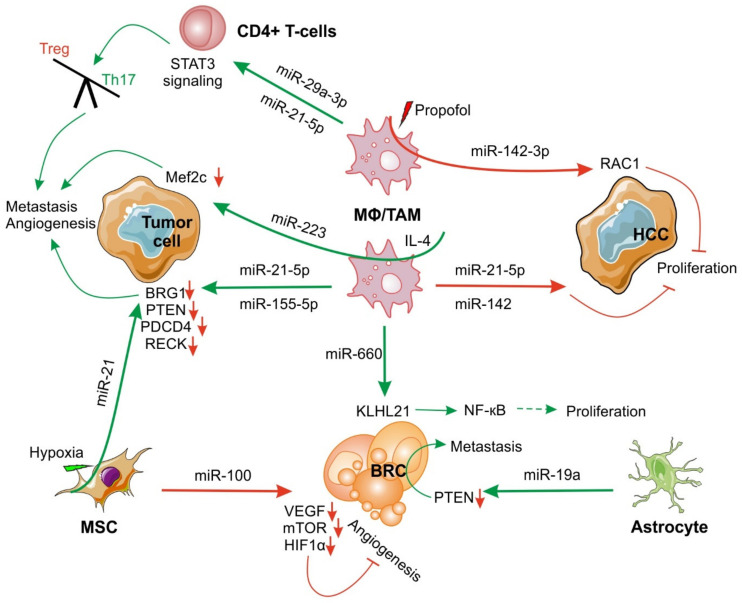
Stroma exosome-derived miRs in the tumor microenvironment. Exosomal miRs are involved in many aspects of tumor immunity in the tumor microenvironment. Stromal miRs provoke tumor cell proliferation and metastasis by regulating angiogenesis and an immunosuppressive tumor microenvironment. See Section 2.2 for details. BRC, breast carcinoma cell; HCC, hepatocellular carcinoma cell; MSC, mesenchymal stem cell. ↓ = Downregulation. Green arrows are pro-tumoral and red arrows are anti-tumoral.

In the context of tumors, the primary task of host immune cells is to control tumor initiation and propagation. More often than not, they successfully perform this task diligently using various means at their disposal, including mediators of intracellular communication such as miRs. Almost all cells, under physiological or pathophysiological conditions, secrete exosomes. Immune cells of tumor stroma such as macrophages, when they are classically activated, secrete exosomes with anti-tumor characteristics. Once tumor-derived factors reprogram macrophages into an alternatively activated phenotype, which is a pro-tumor phenotype, these so-called M2 macrophages produce exosomes with pro-tumoral characteristics. Contents of exosomes, including miRs, determine the outcome of their action on target cancer cells. CD163^+^ colorectal TAMs secrete exosomes containing high levels of miR-21-5p and miR-155-5p. These exosomes are taken up by CRC cells. Exosomal miRs bind to the transcription regulator brahma-related gene-1 (BRG1) coding sequence to downregulate its expression, which is identified as a crucial factor that promotes CRC metastasis (Figure 3) [57]. Similarly, IL-4-activated macrophages release exosomal miR-223, which upon transfer to breast cancer cells, increases their invasiveness in vitro by targeting the Mef2c-β-catenin pathway (Figure 3) [58]. Conversely, it was shown that exosomes, released by monocyte-derived macrophages, contain miR-223 and miR-142. These exosomes are internalized by cocultured HCC cells in a contact-dependent manner, requiring gap junctions. Exosomal miRs then inhibit the proliferation of cancer cells [59]. The anti-tumor drug propofol induces macrophage activation and exosome release. These exosomes derived from TAMs contain miR-142-3p [60], which upon delivery to hepatocellular tumor cells, in a mouse model, inhibits tumor growth and invasion by targeting RAC1 (Figure 3) [60,61]. Furthermore, depletion of miR-142-3p in TAMs reverses the effects of anti-tumor drugs [60]. Cells such as mesenchymal stem cells (MSC) also take part in miR-mediated tumor immunity. MSC transport miR-100 to breast cancer cell via exosomes. Exosomal miR-100 then targets and downregulates the expression of VEGF, mTOR, and HIF-1α (Figure 3). Reduced expression of these angiogenic factors suppresses angiogenesis. Conditioned media of breast cancer cells, stimulated with MSC exosomes, decreases proliferation, migration, and tube formation of human umbilical vein endothelial cells (HUVEC) [62].

One of the salient features of an established tumor is its ability to reprogram host immune cells and tumor stroma to perform pro-tumoral functions, including secretion of pro-tumoral miRs. There are examples in the literature where exosomal cargo of stromal cells supports tumor growth and dissemination. Exosomes derived from activated astrocytes contain miR-19a, which upon delivery to breast cancer cells results in phosphatase and tensin homolog (PTEN) suppression, and thus contributes to metastasis (Figure 3) [63]. In another study, it was shown that exosomal miR-21, secreted by hypoxic mesenchymal stem cells, promotes non-small-cell lung cancer cell mobility, proliferation, and macrophage M2 polarization in vitro and increases tumor growth and intra-tumoral angiogenesis in vivo by inhibiting the expression of PTEN, PDCD4, and RECK (Figure 3) [64]. Cancer-associated fibroblast (CAF)-derived exosomes stimulate the migration of breast cancer cells by inducing Wnt-planar cell polarity (PCP) autocrine signaling [65]. Exosomes from CAFs, containing miR-22, let7a, and miR-125b, suppress oxidative phosphorylation in prostate and pancreas cancer cells and promote glycolysis and glutamine-dependent reductive carboxylation [66]. CAF-derived exosomal miR-181d-5p targets caudal-related homeobox 2 (CDX2), a homeobox protein that is associated with the differentiation of intestinal cells, and a transcription factor homeobox A5 (HOXA5) to accelerate breast cancer progression [67]. Paclitaxel resistance was conferred by CAF-derived exosomal miR-21 that binds to apoptotic protease activating factor 1 (APAF1) in ovarian cancer cells [68]. Similarly, gemcitabine resistance was conferred by macrophage-derived exosomal miR-385 by inducing cytidine deaminase activity in pancreatic cancer cells [69]. CAFs also secrete exosomes containing miRs, such as miR-21, miR-378e, and miR-143, which upon transfer to breast cancer cells increase cancer cell stemness, EMT, anchorage-independent growth, and their invasive capacity [70]. TAMs release exosomes containing miR-660, which could be internalized by breast cancer cells. TAM-derived miR-660 competes with inhibitor kappa B kinase β (IKKβ) to bind kelch-like protein 21 (KLHL21), resulting in activation of NF-κB p65 signaling pathways (Figure 3), which lead to breast cancer progression [71]. In epithelial ovarian cancer, TAMs transfer miR-29a-3p and miR-21-5p to CD4+ T cells through exosomes. These miRs target the STAT3 signaling pathway and skew the Treg/Th17 balance towards Tregs (Figure 3). This Treg/Th17 imbalance generates an immunosuppressive microenvironment, which was associated with metastasis and rapidly growing tumors [72]. Exosomes from bone marrow-derived mesenchymal stem cells (BMSCs) promote lung cancer cell invasion by activating the STAT3 signaling pathway and triggering EMT due to the presence of miR-193a-3p, miR-210-3p, and miR-5100 [73]. Figure 3 summarizes major stroma-derived miRs that play an important role in the tumor microenvironment.

These studies underscore the importance of exosomes in the tumor microenvironment. Though exosomes from tumor cells and stroma are involved in pro- and anti-tumoral effects, their retrovirus-like nature [74,75] is an excellent tool to deliver bioactive molecules. The ability of lateral gene transfer has many implications, even in RNA therapeutics. Their very nature makes them useful and versatile particles for intercellular communication, which may serve as a platform to launch RNA therapeutics, either by chemical modification of the exosome backbone or loading the active ingredient for transport and delivery.

## 3. Non-Exosomal MiRs in Cancer

There are many iterations of non-exosomal miRs, but they all share the common principle that extracellular miRs should be stable and protected from nuclease digestion. The predominantly studied non-exosomal miRs are AGO-bound or RNA-induced silencing complex (RISC)-containing AGO [76,77,78]. However, studies have shown that miRs could be bound to other stabilizing factors such as high- or low-density lipoproteins [17,79,80], or RNA-binding proteins such as nucleophosmin [81].

Contrary to the tumor microenvironment, almost all miRs in plasma of healthy donors could be immunoprecipitated by AGO2 antibodies, suggesting that the majority of plasma-borne miRs are associated with non-exosomal fraction and are free to interact with anti-AGO2 antibodies [12]. However, the existence of pre-miR in non-exosomal fraction is still an open question. AGO2-bound miRs attain a conformation where the phosphodiester backbone appears to be masked from nucleases and is associated with the AGO protein [82]. Furthermore, AGO, once loaded with miR, attain a conformation that is resistant to proteases such as thermolysin [83]. Rapidly dividing tumor cells and cells undergoing apoptosis due to hypoxia or nutrient deprivation release EVs such as exosomes, which contain secretory miRs. However, it has been shown that EVs are stable during freeze–thawing cycles [78], which could be explained by the presence of AGO2 complexes that account for the remarkable stability of exosomal miRs [76,77]. Furthermore, AGO2 also regulates miR sorting. In colon cancer, during exosome biogenesis, AGO2 localizes to multivesicular endosomes, but phosphorylation by KRAS–MEK signaling dissociates AGO2 from endosomes and sorting to exosomes is inhibited [84]. Furthermore, expression and phosphorylation of AGO2 affects the level of several miRs, such as let-7a, miR-100, and miR-320a, in exosomes [84]. However, it is well-known that exosomes contain AGO proteins along with the full battery of miR processing enzymes. Thus, the debate is still open as to whether non-exosomal miRs, isolated in complex with AGO proteins, are extra-exosomal or just the result of an isolation procedure that disrupts the exosome. Furthermore, it is not known if AGO- or RISC-bound miRs are non-exosomal or attached to the external surface of exosomes. Nevertheless, the sheer stability of the so-called non-exosomal miRs in biological fluids makes them an attractive target of cancer therapy.

Not all non-exosomal miRs in the tumor microenvironment are necessarily protected or show the comparable miRome profile to exosomal miRs or miRome. It has been shown that AGO-bound non-exosomal miRs are subjected to degradation based on the exposed 3′ end of miRs, which may be due to the mutation in the AGO protein [85]. These mutations may contribute to cancer progression by altering the half-life of certain miRs. Non-exosomal miRs may be sequestered by higher-order protein complexes, such as AGO2-RISC, that mask the miR in such a way that it is protected against RNase activity and inhibits the base pairing with a target antagomir.

Tumor-derived non-exosomal miRs are scarcely reported in the literature, partly due to difficulty in isolating them or due to the major focus on EV-derived miRs. However, in biological fluids, there is a variety of miRs that are not encapsulated in EVs. One of the most remarkable examples is miR-375. miR-375 has been shown to be highly expressed in breast cancer cells and released upon undergoing apoptosis. This tumor-derived miR was shown to be associated with LDL (low-density lipoprotein). In the tumor microenvironment, LDL was taken up by macrophages via CD36 [17]. The non-exosomal nature of miR-375 is also corroborated by other studies in different biological fluid. It was shown that miR-375 is exclusively associated with LDL and could be isolated as LDL-bound [79] or AGO2-bound miR [12] in human plasma.

The extracellular miR landscape is quite heterogeneous and the binary nature of exosomal and non-exosomal miR is context-dependent (Table 1). Several miRs have been identified in both exosomal and non-exosomal fractions in a context-dependent manner. One of the candidates of this phenomenon is miR-375. We noted that this miR is released by apoptotic breast cancer cells in LDL-bound fractions as this miR was digested during RNase treatment of conditioned media from breast cancer cells [17]. Interestingly, miR-375 is also detected in exosome fractions of human plasma of normal and familial hypercholesterolemia patients [79] and patients with metastatic rectal cancer [86]. However, a substantial portion of miR-375 is also detected in the non-exosomal fraction [79]. Similarly, miR-200c is shown to be present in both exosomal and non-exosomal fractions in the human plasma [79]; however, we noted that it is exclusively present in human breast cancer cell-derived exosomes and internalized by TAMs via CD36 [87]. Inversely, miR-382 is exclusively localized in the non-exosomal fraction and bound with LDL in human plasma, but is reported to be present in CAF-derived exosomes in oral squamous cell carcinoma [88]. Furthermore, there are miRs which are nearly equally distributed between exosomal fractions and non-exosomal fractions, such as miR-106a, miR-135a, and miR-425. In the same patients, even the non-exosomal fractions of the same miRs distribute differently with distinct miR-carriers, such as miR-134, miR-223, miR-339-3p, and miR-766, which are present in both HDL (high-density lipoprotein) and LDL fractions of human plasma [79]. Therefore, for RNA therapeutics of cancer, the clear demarcation of exosomal or non-exosomal miR should be made before selecting any candidate miR for a particular cancer type, and knowledge acquired from other cancer types may not be directly translated without prior investigations. Furthermore, it is likely that the therapeutic delivery of miR mimetics in exosomal or non-exosomal composition might lead to undesired side effects due to preferential uptake of these moieties by cancer cells depending upon the type of cancer, localization, and the stage of cancer.

## 4. Extracellular miR-Based RNA Therapeutics

When the first oncomiRomes were analyzed from healthy tissue vs. cancer samples, it was surprising that miRomes were better at predicting the cancer type and stage than transcriptome profiles [89,90]. RNA therapeutics regulating miR expression is a very attractive option for various reasons, including specificity. It is possible to determine all putative target binding sites of a particular miR in silico, which is not possible for a pharmacological drug due to undermined target conformation in different cell types and in different cellular compartments (e.g., oncoenzyme inhibition). Furthermore, to be able to regulate oncomiRs, very specific miR mimics or antagomirs could be designed. With the advent of a superior delivery method for small RNAs (e.g., SARS-CoV-2 mRNA vaccine [2,91]) and recent developments in the chemistry of the delivery vehicles, ‘miR mimetics’ could be used to alter ‘oncomiRome’. A seminal study to achieve this objective was first reported back in 2005 by Krützfeldt et al. [92]. Intravenous administration of antagomirs against miR-16, miR-22, miR-192, and mR-194 led to a remarkable, long-term reduction of their target mRNAs in various tissues. Although, antagomirs could not pass the blood–brain barrier, but tissue-specificity was lacking. These chemically stable antagomirs could survive for about two weeks in vivo [93,94], whereas stable expression by plasmid vector of hairpin RNA can be achieved [95,96]. Alternatively, overexpression of oncomiR target sequences was shown to attenuate oncomiR function by titrating the oncomiR away from endogenous targets [97,98], suggesting that long-term competitive inhibition of oncomiRs is possible in a clinical scenario. However, AGO2, a key component of RISC, is predicted to prefer double-stranded duplexed miR, whereas extracellular miRs are single-stranded and may load differentially onto AGO-RISC complex or might exploit other AGO family members to bring about gene expression regulation. This aspect needs further clarification in order to mimic and exploit endogenous oncomiRs in clinical settings.

**Figure 4:**
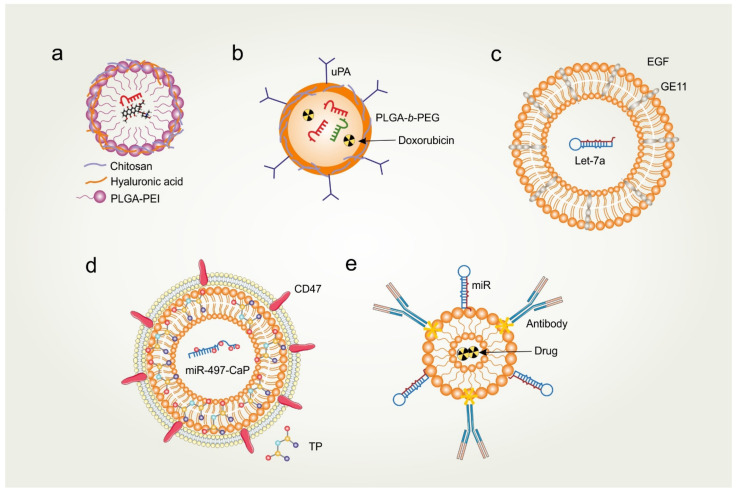
Extracellular miR-based RNA therapeutics. (**a**) A hyaluronic acid (HA)-chitosan decorated or HA-polyethylenimine-poly(d,l-lactide-co-glycolide) (PEI-PLGA) nanoparticle (NP) system developed for targeted co-delivery of doxorubicin and miR-34a or miR-542-3p. (**b**) AntagomiRs loaded to urokinase plasminogen activator (uPA)-peptide and polylactic-co-glycolic acid-polyethylene glycol (PLGA-*b*-PEG) polymer NPs for targeted delivery of antagomiR-10b and antagomiR-21 to uPAR+ cancer cells. The NPs can also be loaded with anti-cancer drugs such as doxorubicin. (**c**) Modified exosomes express the transmembrane domain of platelet-derived growth factor receptor fused to the GE11 peptide or EGF on their surface to deliver Let-7a to EGFR-expressing cancer cells in vivo. (**d**) Hybrid nanoparticles (HENPs) made of synthetic liposomes with encapsulated triptolide (TP) fused with cancer exosomes with CD47 for targeted delivery. Calcium phosphate (CaP)-adsorbed miR-497 incorporated into the NPs. (**e**) Synthetic exosomes designed to deliver adjuvant therapy of miR mimetics with anti-cancer drugs. The surface of these exosomes can be coated with adsorbed miR mimic/antagomiR and an antibody targeting a specific antigen of target cells.

Cancer cell-specific targeting of RNA therapeutic molecules would severely reduce side effects and enhance the therapeutic efficacy. There are approaches which have been exploited for targeted delivery of miR mimetics. It has been shown that hyaluronic acid (HA)-chitosan nanoparticles delivered tumor suppressor miR-34a and doxorubicin (DOX) to triple-negative breast cancer cells in vitro and in vivo by targeting the HA receptor, which is overexpressed in breast cancer (Figure 4a) [99]. This inhibited tumor growth by downregulating the anti-apoptotic proto-oncogene Bcl-2 and targeting Notch-1 signaling. Similarly, HA-coated polyethylenimine-poly(d,l-lactide-co-glycolide) nanoparticles successfully delivered miR-542 and DOX, with improved targeting and increased uptake [100]. Restoration of anti-tumor miR-542 potentiated the apoptotic potential of DOX by activating p53 and inhibiting survivin expression. Furthermore, urokinase plasminogen activator receptor (uPAR)-targeted delivery of antigomiR-21 and antagomir-10b in polylactic-co-glycolic acid (PLGA)-based nanoparticles substantially reduced breast tumor growth (Figure 4b) [101]. This FDA-approved drug delivery method targeted oncomiRs and provided a platform for breast cancer therapy [101,102]. In another study, let-7a was encapsulated in epidermal growth factor (EGF), peptide-conjugated exosomes to target EGFR-expressing breast cancer cells. The delivery of this miR by exosomes, fused with the synthetic peptide GE11, inhibited tumor growth in mice (Figure 4c) [103]. These miR vehicles can also be used for adjuvant therapy, including nucleic acid drugs for cancer treatment. Furthermore, the specificity of the miR vehicles can be enhanced by coating the surface with specific antibodies or ligands against the receptor of target cancer cells. These approaches have merit over conventional adjuvant therapy, such as low risk of drug resistance, promotion of apoptosis and autophagy, suppression of angiogenesis, inhibition of the expression of efflux transporters, and reversion of EMT [104]. By specifically targeting oncomiRs with antagomiRs or restoring the expression of tumor-suppressor miRs, it is possible to sensitize cancer cells to adjuvant chemotherapy drugs. The approach could be further refined by combining multiple miRs to broadly target oncogenic pathways. Recently, a new hybrid nanoplatform, miR497/TP-HENPs, was developed that is composed of exosomes from ovarian cancer cells, liposomes modified by the target cRGD peptide, the chemotherapeutic drug triptolide as the cargo, and miR-497 adsorbed on the surface of the nanoparticles (Figure 4d). These hybrid nanoparticles target tumor cells through tumor-derived exosomes and synthetic cRGD-targeting peptide. In the tumor microenvironment, cleaved nanoparticles release miR-497 and triptolide, which synergistically inhibit PI3K/AKT/mTOR signaling pathways and deplete glutathione to elevate intracellular reactive oxygen species (ROS). Ultimately, this results in tumor cell death and overcomes drug resistance [105]. Nevertheless, though this co-delivery platform holds promise as a better therapeutic modality for the treatment of cancer, it requires further investigation.

Another way to exploit tumor-derived exosomes for targeted miR memetic delivery was recently reported in the literature. The authors developed anti-exosomes antibody-oligonucleotide complexes (ExomiR-Tracker) that exploit tumor exosomes. ExomiR-Tracker binds to the surface of an exosome and hijacks it to carry the therapeutic miR payload and gain entry into target cells. 9-mer of D-arginine enhances the endosomal escape of the anti-miR oligonucleotides, thereby executing the therapeutic miR memetic action [106]. It is noteworthy that miR memetics transported via exosomes might be protected within the acidified tumor microenvironment, which otherwise confers drug resistance. In fact, tumor microenvironmental acidity has been shown to enhance exosomal targeting to and uptake by target tumor cells [107,108]. However, it should also be noted that the delivery of miR mimic or antagomiR to target cells requires miR mimetics to escape the endosomal compartments and reach the cytoplasm, where they can be loaded onto RISC and bind to target mRNAs or miRs. Unfortunately, it has been reported that less than 5% of the delivery payload reaches the cytoplasm [109]. The targeted delivery of miR mimetics and uptake by target cells (tumor or stroma) could be enhanced by the conjugation of various agents, such as peptides, α-tocopherol, cholesterol, antibodies, and CpG oligos (Figure 4e) [110]. Alternatively, overexpression of miR mimetics in stromal cells could also be exploited for anti-tumor therapy [111].

## 5. Conclusions

Targeting a single miR for therapeutic purposes has its limitations as a single miR might be insufficient for clinical purposes due to a variety of related oncomiRs and their multiple targets. This degeneracy of miR action along with their stability poses the biggest challenge in RNA therapeutics of cancer [112,113]. Similarly, there are reports that a 2ʹ-OMe-modified angatomiR-93 was able to inhibit other miR-106bs of the same family, despite a slight preference for the cognate target [114]. We have discussed further limitations in our earlier review [115]. RNA therapeutics of cancer should weigh the effectiveness over non-specific side effects of miR mimics or antagomirs. It was proposed that miR mimics or antagomirs are highly specific and thus discriminate similar miRs [116,117]. However, side effects such as non-specific targeting of mimics or antagomiRs are likely unavoidable at high doses. Supraphysiological doses of mimics or antagomirs may saturate the endogenous miR-processing machinery that might hamper endogenous miR-regulatory networks and may have deleterious effects in cells [118]. miR-targeted RNA therapeutics need further refinement, such as reduced toxicity of the carriers [112], increased accuracy, and control, to be able to transit from pre-clinical to clinical approaches. An alternate approach to improve specificity is to target the pre-miRs with antagomiRs or siRNA strategies [119,120,121]. Short-peptide nucleic acids that bind double-stranded RNA could be used to regulate pre-miRs in vivo [122,123,124,125,126]. Expression or inhibition of miRs can therefore be combined with adjuvant therapies such as cytotoxic drugs. One of the earlier examples of this approach is the treatment of antagomiR-21 together with a secreted form of tumor necrosis factor-related apoptosis-inducing ligand (S-TRAIL), which completely abolished glioblastoma cells [127].

Studies using exosomes as a source of miRs should be interpreted in the context of their origin, localization, target cells, and pathologies that are involved. Exosomes not only contain regulatory nucleic acids such as miRs but are also enriched with immunomodulatory proteins such as CSF-1, CCL2, FTL, TGFβ, etc. [28]. These immunomodulatory factors might have potentiating or opposing effects on the miR activity. The protein content of exosomes may be functionally more important than their miR repertoire. One study suggests that exosomes derived from Lewis lung cancer cells transfer EGFR to host macrophages, which reduces the production of type I interferons and macrophage-mediated antiviral immunity [128]. Furthermore, the biochemical state of the parent cell, such as nutrient deprivation or hypoxia, also determines the effect of exosomes, as it was shown that exosomes derived from hypoxic tumor cells enhance mitochondrial OXPHOS in macrophages [28].

## 6. Future Perspective

The notion that miRs are reliable diagnostic and prognostic markers is based on the premise that a substantial number of miRs can be detected in the plasma of cancer patients. However, the concentration of miRs in plasma is in the range of about 5 pM [129], which is more than 5-fold less than the concentrations considered effective for intracellular functions [130], or ~1000 times higher for inter-target pool competition in the cytoplasm [131]. Although, it has been shown that the biological fluid containing miRs, when exposed to target cells, provokes miR target gene regulation. However, it is still not clear whether the levels in extracellular fluid, such as plasma, reflect the micro-milieu of the tumor microenvironment. A comprehensive database should be established that catalogs the miRome in a particular tumor microenvironment that might aid in the identification of miRs with high fidelity as a diagnostic marker and therapeutic target. Furthermore, accumulating evidence suggests that miRs can also be found and perform their non-canonical functions in the nucleus [132,133,134,135], though their loading to AGO2/RISC may differ in the nucleus compared to the cytoplasm [136]. Nevertheless, miRs can be colocalized with 28S rRNA in the nucleolus and exported with a functional ribosome and their target mRNAs from the nucleus to the cytoplasm [137]. This implies that miRs can function in nucleolar processes and leave the nucleolus to regulate downstream events, such as protein translation at the elongation stage. Furthermore, it was shown that miRs mediate transcriptional gene silencing or activation and post-transcriptional gene activation in the nucleus by directly binding to RNA transcripts or interacting with the promotor or enhancer regions of target genes [135,138,139,140,141]. These non-canonical functions of miRs should be taken into consideration when proposing them as a target in bench-to-bedside approaches.

Some anti-cancer compounds alter the expression of the oncomiRome [142] and miRs also affect drug sensitivity [93,143]. Since the majority of treatment regimens involve adjuvant therapy, it is imperative to study the crosstalk of anti-cancer adjuvant therapy with RNA therapeutics to be able to predict treatment outcomes. Furthermore, by modulating multiple oncomiRs simultaneously, such a miRome-modifying approach may be much more effective for therapy than strategies that aim to regulate a single miR.

It has been shown that the administration of mouse- or human-derived exosomes, in low doses, does not evoke strong immune responses [144,145]. Hence, therapeutic consideration of whole-exosome-containing therapeutic miRs or antagomirs may also be an option, as was previously performed [146]. Plasma or blood transfusions, containing millions of exosome particles, have been performed for decades without an exosome-associated immune response. Although exosomes can cross the blood–brain barrier [147,148], it has been shown that the organotrophic uptake of the exosomes is dependent on the integrin clusters on an exosome [149], which impart cell specificity, and as a result, exosomes are not taken up by all cell types in vivo [150]. Furthermore, context-dependent immune responses against exosomes cannot be ruled out and should be considered in therapeutic settings.

Targeting small RNAs such as miRs should also take into consideration that the expression profile of lncRNA is not affected. It has been shown that lncRNA regulates miR expression [151]. Furthermore, horizontal transfer of lncRNA may also affect target miR expression, such as in the case of sunitinib chemoresistance of RCC. It was shown that CAF-derived exosomes contain lncRNA activated in RCC with sunitinib resistance (lncARSR). This lncRNA competitively binds to miR-34 and miR-449 and neutralizes their ability to downregulate the expression of their target genes AXL and c-MET, respectively. Relieved expression of these tyrosine kinases confers resistance to sunitinib treatment [152].

## Figures and Tables

**Table 1:** Exosomal and non-exosomal miRs in the tumor microenvironment.

Tumor Cell-Derived miRs		
miRs	Cancer/Cell Type	References
miR-212-3p	Pancreatic cancer	[33]
miR-21-5p, miR-200a	CRC	[34]
miR-222-3p	EOC	[35]
miR-182-5p	Glioblastoma	[36,37]
miR-1246	Glioma	[38]
miR-23a, miR-1247-3p	HCC	[39,42]
miR-122, miR-149, miR-181a, miR-181b, miR-1387-3p	Melanoma	[40]
miR-24, miR-92a	Liposarcoma	[41]
miR-21, miR-29a, miR-499a-5p, miR-192	Lung cancer	[43,50]
miR-125b, miR-130, miR-155	Prostate cancer	[45,49]
miR-22, miR-200, miR-105	Breast cancer	[46,51,52]
miR-23a	Lung adenocarcinoma	[47,48]
miR-663b	Cervical cancer	[53]
miR-7-5p	Bronchial epithelial cells	[54]
miR-1290, miR-155	Gastric cancer	[55,56]
**Stromal miRs**		
miR-21-5p, miR-155-5p	Colorectal TAMs	[57]
miR-223, miR-142, miR-385	Macrophages	[58,59,69]
miT-142-3p	HCC TAMs	[60,61]
miR-100, miR-21, miR-193a-3p, miR-210-3p, miR-5100	MSC	[62,64,73]
miR-19a	Astrocytes	[63]
miR-22, let7a, miR-125b, 181d-3p, miR-21, miR-378e, miR-143	CAFs	[66,67,68,69,70]
miR-660	Breast cancer TAMs	[71]
miR-29a-3p, miR-21-5p	EOC TAMs	[72]
**Non-exosomal miRs**		
miR-375	Breast cancer	[12,79,86]
miR-382	Squamous cell carcinoma	[88]
miR-134, miR-223, miR-339-3p, miR-766	Breast cancer, plasma	[79]

## Data Availability

No new data were created or analyzed in this study. Data sharing is not applicable to this article.
